# Serum sST2 and MR-ProADM in pediatric acute rheumatic fever: association with cardiac involvement and clinical risk stratification

**DOI:** 10.3389/fped.2026.1852093

**Published:** 2026-05-29

**Authors:** Ecem Ipek Altınok, Emine Yurdakul Ertürk, Tülin Bayrak, Ceren Yapar Gümüş, Ahmet Bayrak, Taner Kasar

**Affiliations:** 1 Department of Pediatrics, Faculty of Medicine, Ordu University, Ordu, Türkiye; 2 Department of Medical Biochemistry, Faculty of Medicine, Ordu University, Ordu, Türkiye; 3 Department of Pediatric Cardiology, Faculty of Medicine, Ordu University, Ordu, Türkiye

**Keywords:** acute rheumatic fever, biomarker, cardiac involvement, MR-proADM, sST2

## Abstract

**Background:**

Acute rheumatic fever (ARF) remains a major cause of acquired heart disease in children, particularly in low- and middle-income countries. Cardiac involvement is the main determinant of long-term morbidity and mortality; however, early evaluation relies heavily on echocardiography, which may not always be readily accessible. Therefore, there is a need for adjunctive biomarkers to support clinical assessment and risk stratification.

**Methods:**

This case–control study included 38 pediatric patients diagnosed with ARF and 38 age- and sex-matched healthy controls. Serum levels of soluble suppression of tumorigenicity-2 (sST2) and mid-regional pro-adrenomedullin (MR-ProADM) were measured using enzyme-linked immunosorbent assay. Cardiac involvement was assessed by echocardiography and further categorized as mild or no involvement vs. moderate-to-severe involvement. Nonparametric tests, receiver operating characteristic (ROC) analysis, and multivariate logistic regression were performed.

**Results:**

Both sST2 and MR-ProADM levels were significantly higher in patients with ARF compared to controls (*p* < 0.001 and *p* = 0.046, respectively). sST2 demonstrated good discriminative performance for distinguishing ARF from controls (AUC: 0.81), whereas MR-ProADM showed moderate performance (AUC: 0.67). When evaluating clinically relevant cardiac involvement, both biomarkers showed acceptable discriminative ability (AUC: 0.732 for sST2 and 0.707 for MR-ProADM). In multivariate analysis, sST2 was independently associated with moderate-to-severe cardiac involvement (OR: 1.02, 95% CI: 1.002–1.042, *p* = 0.033), while MR-ProADM showed a borderline association.

**Conclusion:**

sST2 and MR-ProADM are elevated in pediatric ARF and reflect systemic inflammatory and cardiovascular processes. While they are not substitutes for echocardiographic evaluation, sST2 in particular may provide additional information in identifying patients with more clinically significant cardiac involvement. These biomarkers may serve as supportive tools for clinical assessment and early risk stratification, especially in resource-limited settings.

## 1 Introduction

Acute rheumatic fever (ARF) remains a leading cause of acquired heart disease among children in low- and middle-income countries (1, 2). Cardiac involvement, primarily affecting the cardiac valves, is the most important determinant of long-term morbidity and mortality.

Early identification of cardiac involvement is essential for timely referral and management; however, current diagnostic approaches rely mainly on echocardiography, which may not always be accessible in resource-limited settings (1). Therefore, there remains a need for reliable biomarkers to support early clinical assessment and risk stratification of cardiac involvement in ARF (3, 4).

Circulating biomarkers reflecting myocardial stress and vascular dysfunction have gained increasing attention. Soluble suppression of tumorigenicity-2 (sST2) is associated with myocardial strain, inflammation, and fibrosis, whereas mid-regional pro-adrenomedullin (MR-ProADM) reflects endothelial function and microvascular regulation (5, 6).

Particularly, their potential to identify patients with more clinically significant cardiac involvement remains to be clarified.

This study aimed to evaluate the association of sST2 and MR-ProADM with cardiac involvement and to explore their potential role as adjunctive biomarkers in clinical risk stratification in pediatric acute rheumatic fever.

## 2 Materials and methods

### 2.1 Study design and population

This case–control study was conducted between March 2023 and March 2025 at Ordu University Training and Research Hospital. The study included 38 pediatric patients (<18 years) diagnosed with acute rheumatic fever (ARF) and 38 age- and sex-matched healthy controls. Controls were selected using a simple random sampling method from children attending the outpatient clinic for routine evaluation.

The diagnosis of ARF was established according to the revised Jones criteria. The control group consisted of children aged 5–18 years without evidence of acute infection, chronic disease, or inflammatory conditions.

The study was approved by the Clinical Research Ethics Committee of Ordu University (Approval No: 2024/76) and conducted in accordance with the Declaration of Helsinki. Written informed consent was obtained from the parents or legal guardians of all participants. The diagnosis of ARF was established according to the revised Jones criteria. The control group consisted of children aged 5–18 years without evidence of acute infection, chronic disease, or inflammatory conditions, who were evaluated in the outpatient clinic.

The study was approved by the Clinical Research Ethics Committee of Ordu University (Approval No: 2024/76) and conducted in accordance with the Declaration of Helsinki. Written informed consent was obtained from the parents or legal guardians of all participants.

### 2.2 Cardiac evaluation

Cardiac involvement was assessed using transthoracic echocardiography performed by a pediatric cardiologist. Cardiac involvement was defined as the presence of pathological valvular regurgitation consistent with rheumatic valvulitis, primarily affecting the mitral and/or aortic valves, in accordance with established echocardiographic criteria.

For analytical purposes, patients were first classified according to the presence or absence of cardiac involvement. In addition, cardiac involvement was further categorized based on echocardiographic severity into mild or no significant involvement and moderate-to-severe involvement.,

### 2.3 Laboratory measurements

Blood samples were obtained at the time of diagnosis, prior to initiation of anti-inflammatory or antibiotic treatment. Routine laboratory parameters, including white blood cell count (WBC), neutrophil count, lymphocyte count, platelet count, and C-reactive protein (CRP), were recorded.

Serum samples were centrifuged and stored at −90 °C until analysis. Concentrations of soluble suppression of tumorigenicity-2 (sST2) and mid-regional pro-adrenomedullin (MR-ProADM) were measured using commercially available enzyme-linked immunosorbent assay (ELISA) kits, in accordance with the manufacturers’ instructions.

### 2.4 Statistical analysis

Statistical analyses were performed using SPSS software (IBM SPSS Statistics for Windows, version 26.0; IBM Corp., Armonk, NY, USA). The distribution of continuous variables was assessed using the Shapiro–Wilk test. As the data were not normally distributed, nonparametric methods were applied.

Continuous variables were expressed as median and interquartile range (IQR), while categorical variables were presented as frequencies and percentages. Between-group comparisons were performed using the Mann–Whitney U test for continuous variables and the chi-square test for categorical variables.

To evaluate independent predictors of moderate-to-severe cardiac involvement, multivariate logistic regression analysis was performed including sST2, MR-ProADM, and CRP as covariates. Variables were selected based on clinical relevance and to minimize the risk of overfitting. Results were reported as odds ratios (ORs) with 95% confidence intervals (CIs).

Receiver operating characteristic (ROC) curve analysis was conducted to assess the discriminative performance of sST2 and MR-ProADM. The area under the curve (AUC), sensitivity, specificity, and optimal cut-off values were calculated.

A *p*-value < 0.05 was considered statistically significant.

## 3 Results

### 3.1 Demographics

A total of 38 pediatric patients with acute rheumatic fever (ARF) and 38 age- and sex-matched healthy controls were included in the study. The two groups were comparable in terms of age and sex distribution (*p* > 0.05).

### 3.2 Laboratory findings

Patients with ARF showed significantly higher inflammatory marker levels compared to controls. Additional hematological findings, including leukocyte count, neutrophil count, and platelet count, are presented in Supplementary Table S1. Median CRP levels were markedly elevated in the ARF group (26.9 mg/L vs. 2.5 mg/L, *p* < 0.001).

**Figure 1:**
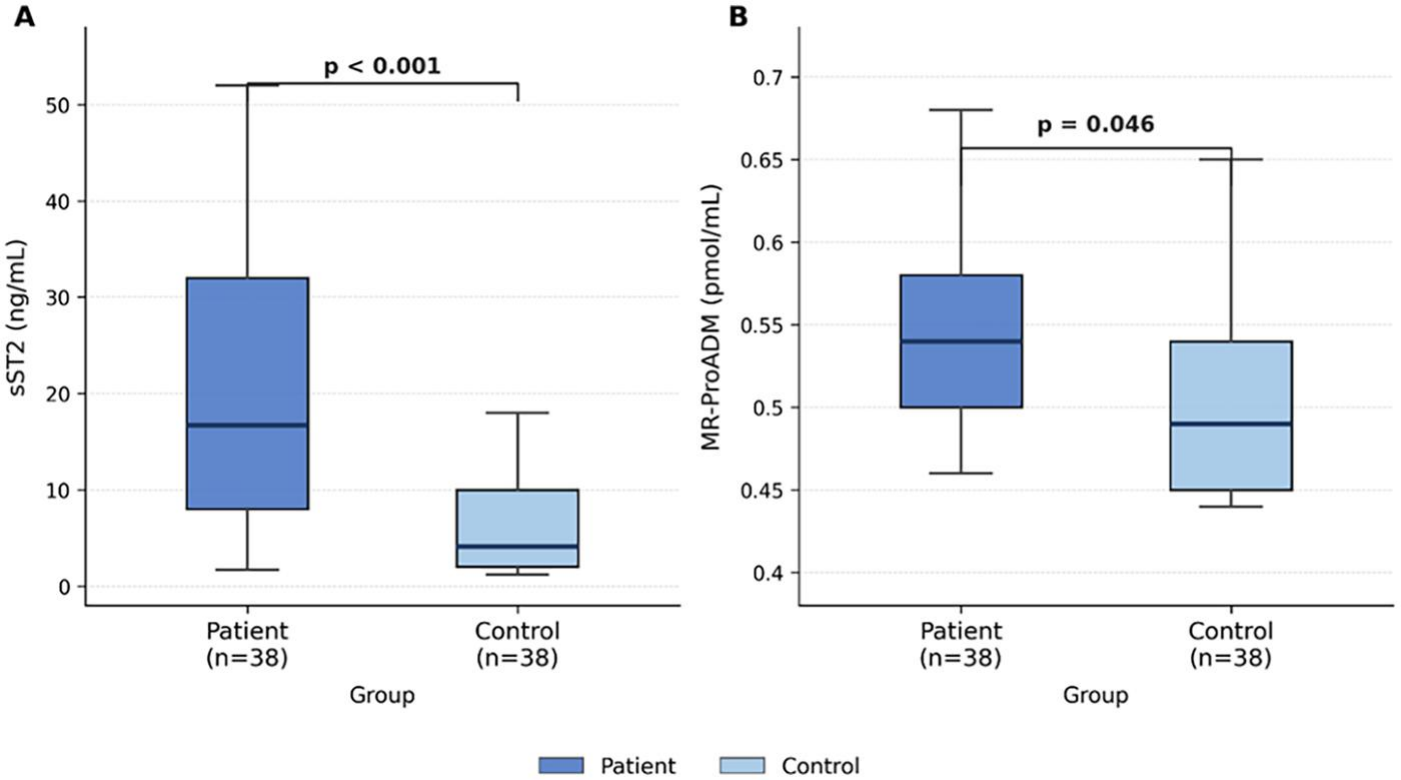
Serum sST2 and MR-ProADM levels in patients with acute rheumatic fever and healthy controls. Box-and-whisker plots showing median and interquartile range. sST2 and MR-ProADM levels were significantly higher in the ARF group compared to healthy controls (*p* < 0.001 and *p* = 0.046, respectively).

Serum levels of sST2 and MR-ProADM were also significantly higher in patients with ARF. Median sST2 levels were 16.7 ng/mL in the patient group compared to 4.1 ng/mL in controls (*p* < 0.001), while MR-ProADM levels were 0.54 pmol/mL vs. 0.49 pmol/mL, respectively (*p* = 0.046). (Table 1, Figure 1).

**Table 1:** Comparison of primary biomarkers and CRP levels between ARF patients and healthy controls.

Parameter	ARF (*n* = 38)	Control (*n* = 38)	*p*-value
CRP (mg/L)	26.9 (10.0–60.0)	2.5 (1.0–5.0)	<0.001
sST2 (ng/mL)	16.7 (8.0–35.0)	4.1 (2.0–8.0)	<0.001
MR-ProADM (pmol/mL)	0.54 (0.50–0.60)	0.49 (0.45–0.55)	0.046

Data are presented as median (interquartile range). Continuous variables were compared using the Mann–Whitney U test. A *p*-value < 0.05 was considered statistically significant.

**Figure 2:**
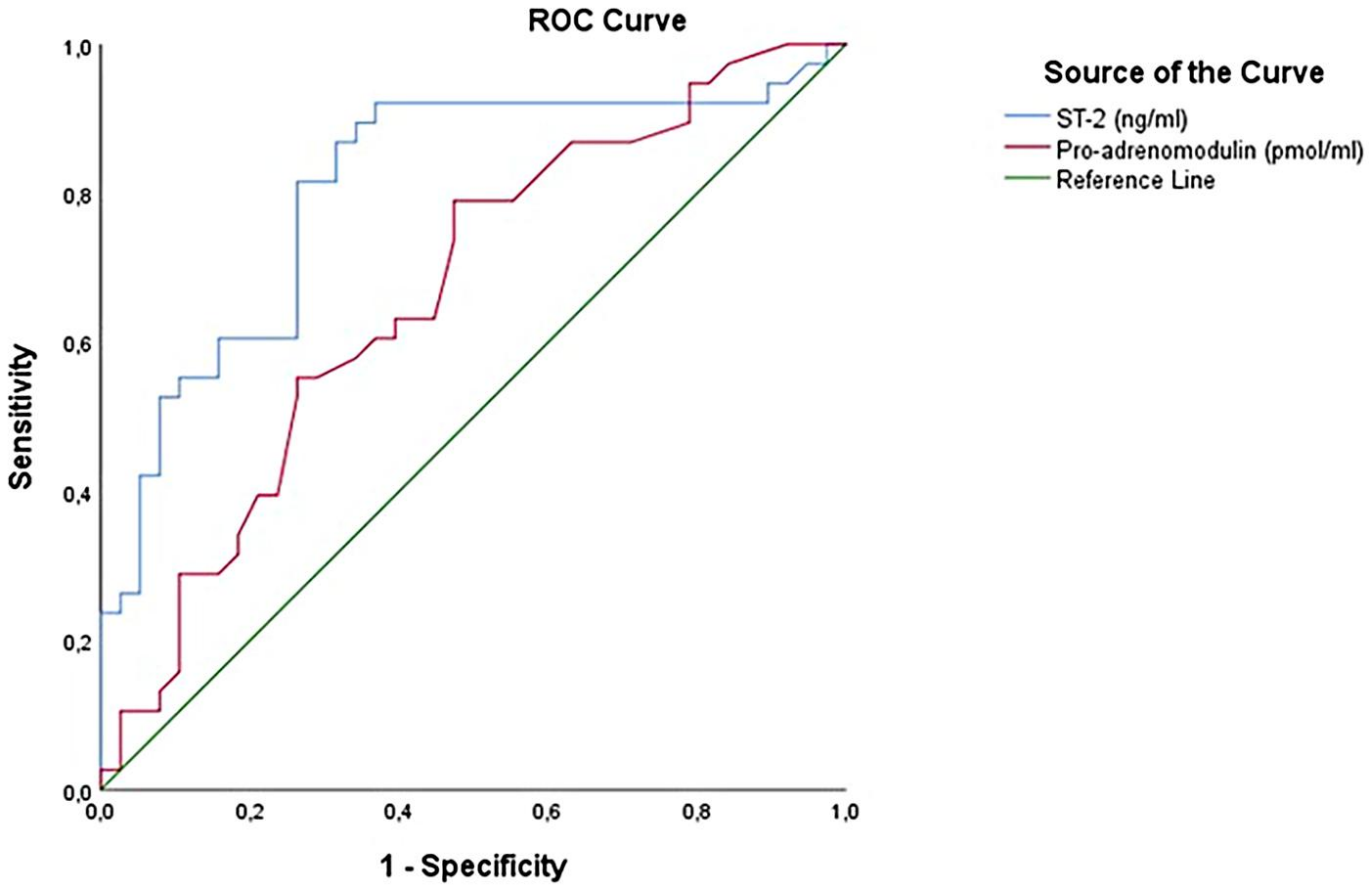
Receiver operating characteristic (ROC) curves of sST2 and MR-ProADM for differentiating ARF patients from healthy controls. The curves show sST2 (red line) and MR-ProADM (blue line). The diagonal line represents no discriminative ability.

ROC curve analysis demonstrated good discriminative performance of sST2 in distinguishing patients with ARF from healthy controls (AUC: 0.81), while MR-ProADM showed moderate discriminative ability (AUC: 0.67) (Table 2, Figure 2).

**Table 2:** ROC analysis of biomarkers for differentiating ARF patients from healthy controls.

Biomarker	AUC	95% CI	*p*-value	Cut-off	Sensitivity	Specificity
sST2 (ng/mL)	0.81	0.70–0.91	<0.001	7.90	73.7%	73.7%
MR-ProADM (pmol/mL)	0.67	0.54–0.79	0.013	0.51	60.5%	60.5%

ROC, receiver operating characteristic; AUC, area under the curve. CI, confidence interval; SE, standard error. Confidence intervals were calculated as AUC ± 1.96 × SE.

### 3.3 Cardiac involvement analysis

Among patients with ARF, cardiac involvement was identified in 34 of 38 patients (89.5%) based on echocardiographic findings, predominantly in the form of valvular regurgitation affecting the mitral and/or aortic valves.

However, when biomarker levels were compared according to the presence of cardiac involvement, no statistically significant differences were observed between groups for either sST2 (17.0 vs. 15.5 ng/mL, *p* = 0.808) or MR-ProADM (0.56 vs. 0.53 pmol/mL, *p* = 0.606).

**Figure 3:**
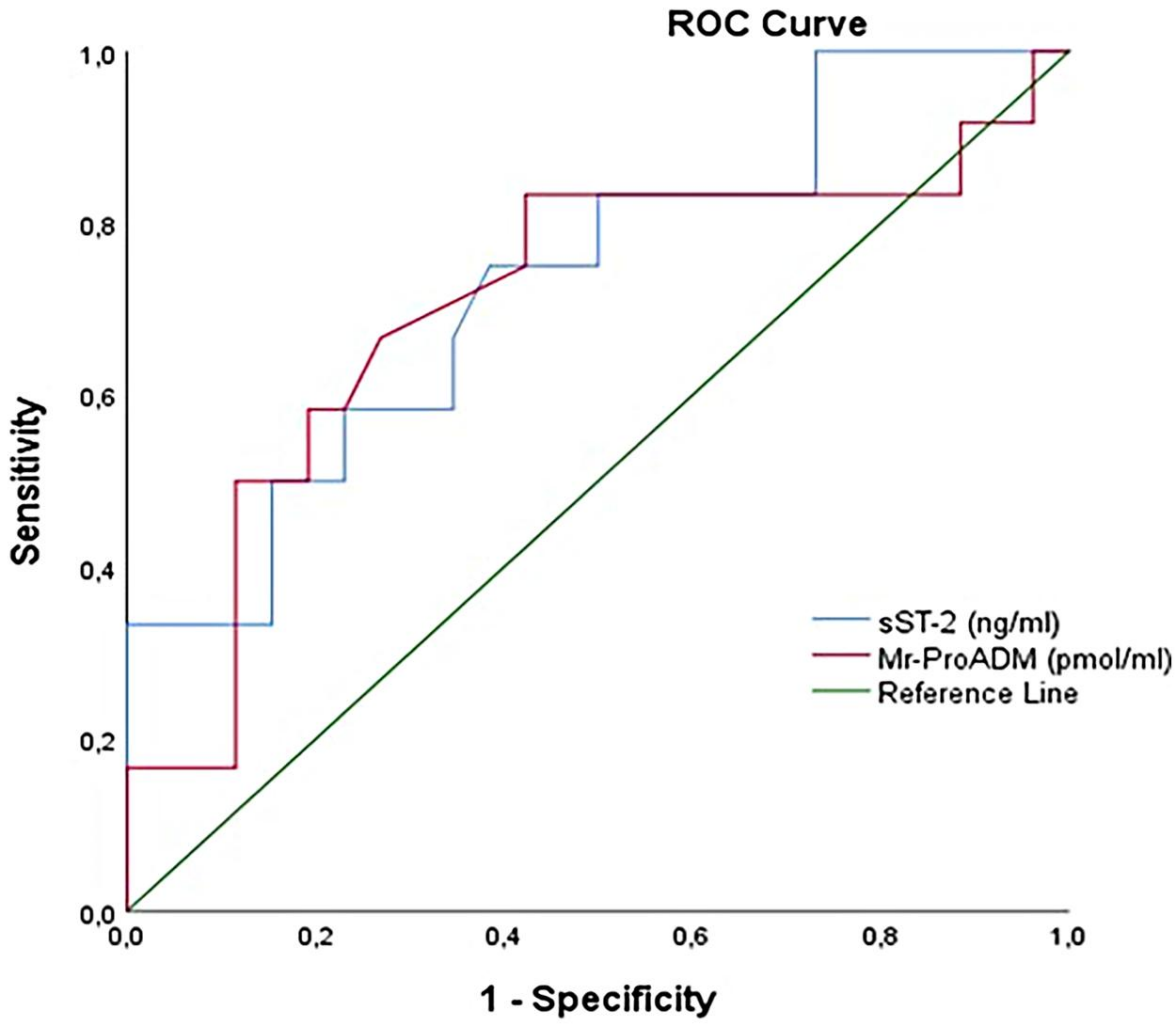
Receiver operating characteristic (ROC) curves of sST2 and MR-ProADM for predicting moderate-to-severe cardiac involvement in patients with acute rheumatic fever. Receiver operating characteristic (ROC) curves demonstrating the diagnostic performance of sST2 (blue line) and MR-ProADM (red line) in predicting moderate-to-severe cardiac involvement. The diagonal green line represents the line of no discrimination. sST2 showed an AUC of 0.732, while MR-ProADM demonstrated an AUC of 0.707, indicating acceptable discriminative ability for both biomarkers.

When focusing specifically on moderate-to-severe cardiac involvement, both biomarkers demonstrated significant discriminative performance. ROC analysis showed that sST2 had an AUC of 0.732 (*p* = 0.005), while MR-ProADM had an AUC of 0.707 (*p* = 0.012), indicating acceptable diagnostic accuracy for identifying patients with more severe disease (Table 3, Figure 3).

**Table 3:** ROC analysis for prediction of moderate-to-severe cardiac involvement.

Biomarker	AUC	Std. Error	*p*-value	Cut-off	Sensitivity (%)	Specificity (%)
sST2 (ng/mL)	0.732	0.071	0.005	17.45	66.7	65.4
MR-ProADM (pmol/mL)	0.707	0.078	0.012	0.54	75.0	57.6

AUC, area under the curve; Std. Error, standard error; Cut-off, optimal threshold determined by Youden index.

Moderate-to-severe cardiac involvement positive (*n* = 12); mild or no cardiac involvement (*n* = 22).

### 3.4 Logistic regression

In multivariate logistic regression analysis evaluating predictors of moderate-to-severe cardiac involvement, sST2 was identified as an independent predictor (OR: 1.02, 95% CI: 1.002–1.042, *p* = 0.033).

MR-ProADM showed a borderline association (OR: 1.29, 95% CI: 0.428–4.031, *p* = 0.072), while CRP was not significantly associated with moderate-to-severe cardiac involvement (*p* = 0.151).

The overall model demonstrated acceptable explanatory power (Nagelkerke R^2^ = 0.367), indicating that approximately 36.7% of the variance in moderate-to-severe cardiac involvement was explained by the included variables (Table 4).

**Table 4:** Multivariate logistic regression analysis for predictors of moderate-to-severe cardiac involvement in patients with ARF.

Variable	Odds Ratio (OR)	95% CI	*p*-value
sST2 (ng/mL)	1.02	1.002–1.042	0.033
MR-ProADM (pmol/mL)	1.29	0.428–4.031	0.072
CRP (mg/L)	1.01	0.997–1.021	0.151

Data are presented as odds ratios (OR) with 95% confidence intervals (CI). The dependent variable was moderate-to-severe cardiac involvement.

## 4 Discussion

Acute rheumatic fever (ARF) remains a major cause of acquired heart disease in children worldwide, particularly in low- and middle-income countries, where delayed diagnosis and limited access to advanced imaging modalities contribute to adverse outcomes (1–3). In this context, there is increasing interest in identifying circulating biomarkers that may assist in the evaluation of disease activity and cardiac involvement.

In the present study, both sST2 and MR-ProADM levels were significantly elevated in children with ARF compared to healthy controls, supporting their association with systemic inflammation and cardiovascular stress in the acute phase of the disease. These findings are consistent with previous reports demonstrating the role of inflammatory and hemodynamic biomarkers in cardiovascular conditions (5–9).

sST2, a biomarker associated with myocardial stress, inflammation, and fibrosis through the IL-33/ST2 signaling pathway, has been widely studied in heart failure and myocardial injury. Elevated sST2 levels have been shown to correlate with adverse cardiac remodeling and clinical outcomes in both adult and pediatric populations (4–7, 10–12). In pediatric settings, sST2 has also been associated with myocardial inflammation in acute conditions such as myocarditis, where it reflects disease severity and early myocardial injury (13). In our cohort, the significant elevation of sST2 in ARF patients suggests that similar mechanisms of myocardial stress and inflammatory activation may be present even in the early stages of rheumatic involvement.

MR-ProADM, a stable surrogate marker of adrenomedullin, reflects endothelial function and microvascular integrity. Previous studies have demonstrated its association with disease severity and vascular dysfunction in conditions such as sepsis, heart failure, and pulmonary hypertension (8, 9, 14). In pediatric cardiovascular disease, MR-ProADM has been linked to hemodynamic impairment and endothelial activation, particularly in pulmonary hypertension associated with congenital heart disease (15). The elevation of MR-ProADM observed in our study supports the presence of early endothelial dysfunction and systemic inflammatory response in ARF. Although MR-ProADM did not retain independent significance in multivariate analysis, its association with endothelial dysfunction suggests that it may still contribute to a broader multimarker approach.

However, when biomarker levels were evaluated according to the presence of cardiac involvement, no significant differences were observed. This finding suggests that although sST2 and MR-ProADM are elevated in ARF, their ability to distinguish cardiac involvement when used alone may be limited. This observation reflects the complex and multifactorial pathophysiology of ARF, in which systemic inflammation, myocardial stress, and endothelial dysfunction may not directly correspond to the degree of echocardiographically detectable valvular involvement.

The apparent discrepancy between the lack of significant differences in group comparisons and the identification of sST2 as an independent predictor in multivariate analysis may be explained by the ability of regression models to account for interactions between variables and adjust for confounding factors. Notably, in our multivariate model, sST2 emerged as an independent predictor of moderate-to-severe cardiac involvement, whereas MR-ProADM and CRP did not retain statistical significance. These findings suggest that sST2 may provide incremental information beyond conventional inflammatory markers and may have potential value as an adjunctive biomarker in clinical assessment. Furthermore, sST2 may reflect subclinical myocardial stress not fully captured by echocardiographic assessment.

In ROC analysis, sST2 demonstrated good discriminative performance in distinguishing ARF patients from healthy controls, while MR-ProADM showed moderate performance. In addition, both biomarkers demonstrated acceptable discriminative ability in identifying moderate-to-severe cardiac involvement. These findings suggest that these biomarkers may be more useful in identifying patients with more clinically relevant cardiac involvement rather than merely detecting its presence.

Importantly, the aim of this study was not to replace echocardiographic assessment or to determine the exact severity of cardiac involvement, but rather to explore whether circulating biomarkers could help identify patients at higher risk of clinically significant cardiac involvement. In this context, these biomarkers should be interpreted as complementary tools that may support early clinical suspicion and prioritization for cardiologic evaluation, particularly in settings where immediate access to echocardiography is limited.

To the best of our knowledge, this study is among the limited number of studies evaluating sST2 and MR-ProADM together in pediatric ARF, and one of the few to explore their potential role in identifying patients with clinically significant cardiac involvement. These findings support the potential value of a multimarker approach combining biomarkers reflecting different pathophysiological pathways.

This study has several limitations. First, the relatively small sample size may have limited the statistical power to detect subgroup differences. In addition, the number of moderate-to-severe cardiac involvement cases was limited, resulting in a relatively low events-per-variable ratio in the multivariate regression model. The events-per-variable ratio remained below the traditionally recommended threshold for logistic regression modelling, and the regression findings should therefore be interpreted cautiously. Second, the single-center design may restrict the generalizability of the findings. Second, the single-center design may restrict the generalizability of the findings. Third, the cross-sectional design precludes assessment of temporal changes in biomarker levels and their relationship with disease progression.

In conclusion, sST2 and MR-ProADM are elevated in pediatric ARF and reflect systemic inflammatory and cardiovascular processes. While their ability to distinguish cardiac involvement is limited when used individually, sST2 appears to provide more consistent information in identifying patients with more clinically significant cardiac involvement. These biomarkers may serve as adjunctive tools to support clinical assessment and early risk stratification, particularly in resource-limited settings. Larger, multicenter, and longitudinal studies are required to further define their role in the management of ARF.

## Data Availability

The raw data supporting the conclusions of this article will be made available by the authors, without undue reservation.
