# Supplementary material for: Serum sST2 and MR-ProADM in pediatric acute rheumatic fever: association with cardiac involvement and clinical risk stratification
Source: Front Pediatr. 2026 May 29;14:1852093. doi: 10.3389/fped.2026.1852093 (PMC13260576; doi:10.3389/fped.2026.1852093)
Supplement: Supplementary file 1 [file Table1.docx]

**Spuplementary** **Table 1. Comparison of Additional Hematological Parameters Between ARF Patients and Healthy Controls**

| **Parameter** | **ARF (n=38)** | **Control (n=38)** | **p-value** |
| --- | --- | --- | --- |
| WBC (×10⁹/L) | 8.9 (7.0–12.0) | 7.8 (6.5–9.0) | 0.001 |
| Neutrophils | 5.4 (4.0–7.5) | 4.0 (3.5–5.0) | 0.001 |
| Platelets | 353 (300–420) | 306 (270–350) | 0.023 |

*Data are presented as median (interquartile range). Continuous variables were compared using the Mann–Whitney U test. A p-value < 0.05 was considered statistically significant.*
